# The Contribution of Mangrove Expansion to Salt Marsh Loss on the Texas Gulf Coast

**DOI:** 10.1371/journal.pone.0125404

**Published:** 2015-05-06

**Authors:** Anna R. Armitage, Wesley E. Highfield, Samuel D. Brody, Patrick Louchouarn

**Affiliations:** 1 Department of Marine Biology, Texas A&M University at Galveston, Galveston, Texas, United States of America; 2 Department of Marine Sciences, Texas A&M University at Galveston, Galveston, Texas, United States of America; 3 Department of Landscape Architecture & Urban Planning, Texas A&M University, College Station, Texas, United States of America; 4 Department of Oceanography, Texas A&M University, College Station, Texas, United States of America; MESC; University of South Alabama, UNITED STATES

## Abstract

Landscape-level shifts in plant species distribution and abundance can fundamentally change the ecology of an ecosystem. Such shifts are occurring within mangrove-marsh ecotones, where over the last few decades, relatively mild winters have led to mangrove expansion into areas previously occupied by salt marsh plants. On the Texas (USA) coast of the western Gulf of Mexico, most cases of mangrove expansion have been documented within specific bays or watersheds. Based on this body of relatively small-scale work and broader global patterns of mangrove expansion, we hypothesized that there has been a recent regional-level displacement of salt marshes by mangroves. We classified Landsat-5 Thematic Mapper images using artificial neural networks to quantify black mangrove (*Avicennia germinans*) expansion and salt marsh (*Spartina alterniflora* and other grass and forb species) loss over 20 years across the entire Texas coast. Between 1990 and 2010, mangrove area grew by 16.1 km^2^, a 74% increase. Concurrently, salt marsh area decreased by 77.8 km^2^, a 24% net loss. Only 6% of that loss was attributable to mangrove expansion; most salt marsh was lost due to conversion to tidal flats or water, likely a result of relative sea level rise. Our research confirmed that mangroves are expanding and, in some instances, displacing salt marshes at certain locations. However, this shift is not widespread when analyzed at a larger, regional level. Rather, local, relative sea level rise was indirectly implicated as another important driver causing regional-level salt marsh loss. Climate change is expected to accelerate both sea level rise and mangrove expansion; these mechanisms are likely to interact synergistically and contribute to salt marsh loss.

## Introduction

Landscape-level shifts in plant species distribution and abundance can fundamentally change the ecology of an ecosystem. These shifts often occur in response to environmental drivers such as climate change, which can cause poleward migration of tropical plant species such as mangroves [1]. In other cases, climate change increases rainfall or atmospheric carbon dioxide concentration, which can facilitate woody encroachment into grasslands [2, 3]. Eustatic and relative sea level rise linked to global warming can cause inland migration of coastal marsh and mangrove species [4]. Species shifts can also be a response to direct anthropogenic alteration of the landscape (e.g., overgrazing [5, 6]) or influences on the control mechanisms (e.g., fire suppression [7, 8]). In most cases, patterns of species shifts are caused by a complex combination of environmental and anthropogenic drivers at landscape-level scales.

Patterns of vegetation shifts in coastal subtropical latitudes where temperate marsh plants and tropical mangrove species coexist are particularly complex. The boundaries between marsh and mangrove habitat fluctuate in response to environmental conditions, as cold-sensitive mangroves die back during freeze events and expand during warm periods, creating a dynamic ecotone [9]. Vegetation composition within this ecotone is further influenced by a complex set of environmental and anthropogenic drivers, such as sea level rise, changes in rainfall, dredging and filling, structural development, shoreline stabilization projects, subsidence, and eutrophication [10, 11]. The wide range of critical ecosystem services provided by coastal habitats [12] suggests that shifts in foundation vegetation species could have substantial implications for local and regional economies.

Recently, the expansion of mangroves into salt marshes has received substantial attention, as it is occurring at many sites in both hemispheres [1]. Mangrove expansion into salt marshes is a coastal case of woody encroachment, where low-stature forbs and grasses are replaced by taller, woody vegetation [13]. The expansion and contraction of mangrove stands occurs naturally on decadal scales in response to disturbance (e.g., fires, hurricanes) and climate (e.g., temperature, rainfall) [14, 15]. This effectively creates dynamic alternate stable states between stands of grasses and forest [9]. Of growing concern among coastal resource managers is the apparent recent acceleration of mangrove expansion [14, 16–20], possibly due to recent climate changes—specifically, the decrease in winter temperature minima, as predicted by many climate change models (e.g., [21]).

On the western Gulf of Mexico coastline, mangroves and marshes co-exist, but high salinity and periodic freeze events have previously limited mangrove expansion [16, 22–25]. There has been a notable increase in mangrove cover in certain bays throughout the Gulf Coast [14, 16, 17, 19]. For example, on Harbor Island (Aransas Bay, Texas), black mangroves (*Avicennia germinans*) more than doubled in area from 1930 to 2004 [20]. These studies clearly demonstrate localized expansion, but it is not yet clear whether mangroves are displacing salt marshes at a larger, regional scale. Therefore, based on the body of relatively small-scale work and broader global patterns of mangrove expansion, we hypothesized that there has been a recent regional-level displacement of salt marshes by mangroves in the western Gulf of Mexico. We focused on the Texas (USA) coastline because recent work has documented a number of “hot spots” of mangrove expansion on the portion of the Gulf of Mexico coast over the last 20 years [16, 19, 20].

## Materials and Methods

### Ethics statement

This study used publicly-available Landsat 5 TM images; no permits are required to obtain or analyze these images. No human or other vertebrate subjects were involved in this study.

We used remotely sensed imagery at a uniquely large, regional spatial scale to test our hypothesis that mangroves are replacing salt marshes on the western Gulf of Mexico coastline. Satellite remote sensing provides a cost-effective approach for regional-scale reconstructions with comparable accuracy to aerial photos [26]. Multiple sensors make repeated return passes that facilitate wetland mapping and provide the ability to conduct land cover change analysis.

**Fig 1:**
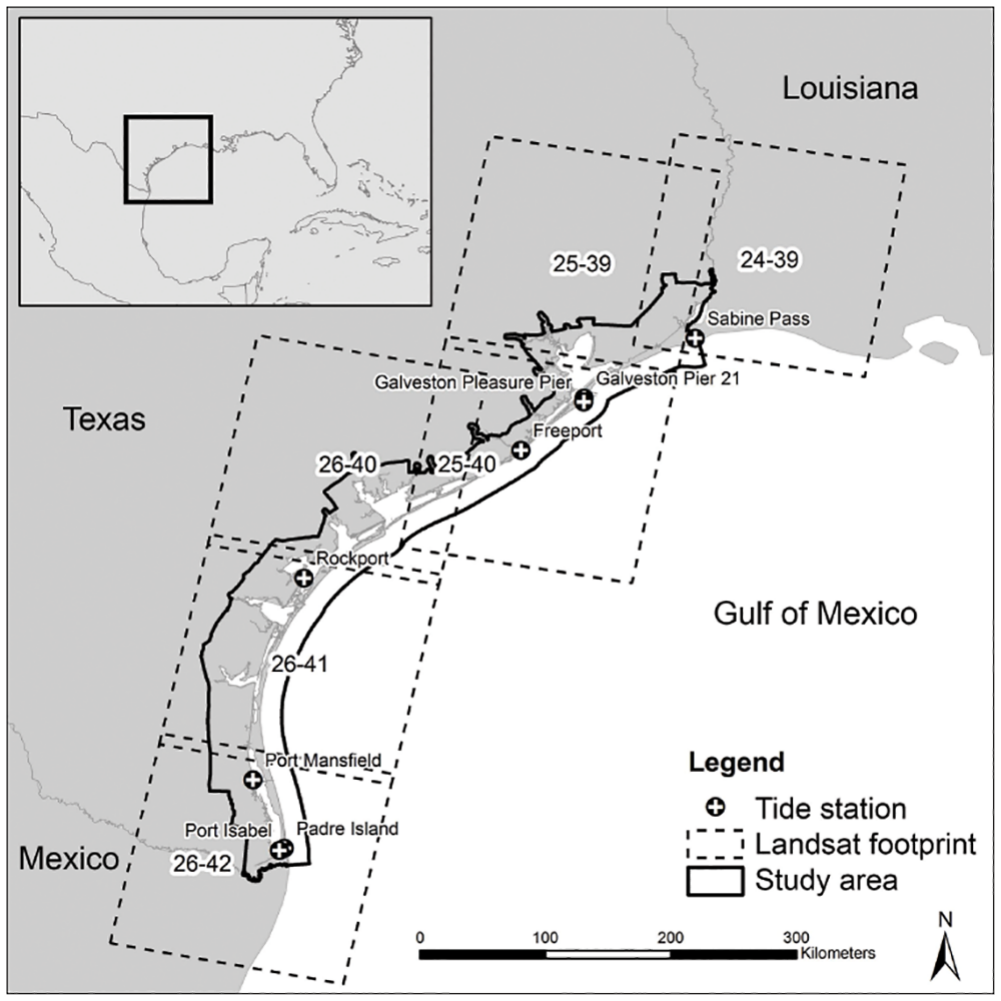
Coastal Zone Management study area and Landsat 5 TM footprints.

Our study area included the entire Coastal Zone Management (CZM) boundary of the State of Texas and is covered by six Landsat images (Fig 1). This large study area covers a total of 26,241 square kilometers, 71% of which is land. Landsat 5 TM images were obtained from USGS for 1990 and 2010 as close to near anniversary in terms of date and water level as cloud cover would allow (Table 1). Water levels in 1990 were not available for all paths, but the subset of available data indicates that for each site, 1990 tidal levels were slightly higher than in 2010, and that tide levels varied among paths (Table 1). However, within each path, the 1990 and 2010 water levels were within < 0.1 m of each other (Table 1).

**Table 1:** Landsat 5 TM image locations, acquisition dates, and water level at time of image acquisition (select stations).

	1990		2010	
Path-Row	Date	Water level (meters above MLLW)	Date	Water level (meters above MLLW)
24–39	5/23	NA	4/28	0.088
25–39	4/28	NA	5/5	0.113
25–40	4/12	0.429	5/5	0.387
26–40	5/29	0.085	3/25	-0.008
26–41	3/18	NA	3/25	0.287
26–42	3/18	0.258	4/26	0.220

NA indicates that water level data were not available for that date.

Image pre-processing steps included calibration to reflectance values and atmospheric correction through dark object subtraction. The data used to train supervised classifications was developed using several different sources. Imagery from 2010 was classified first using training data developed from a combination of 2010 DOQQs from the National Agriculture Imagery Program and seven verified field sites (three mangrove sites near Rockport and four salt marsh sites near Rockport and Galveston, Fig 1). Training data for 1990 was developed using a combination of National Aerial Photography Program (NAPP) imagery from 1989 and National Wetlands Inventory (NWI) data (codes E2E1N for salt marsh and E2SS3N for mangroves). The NWI data was used as an initial guide to locating probable salt marsh, mangrove, and other wetlands. Once located, NAPP imagery was used to confirm and delineate training areas. Approximately 30 sites per scene were used to train the classifications for each land cover class. To improve classification accuracy, we defined the boundaries of the salt marsh/mangrove ecotone and delineated the transition to upland habitats by delimiting contours along elevations optimal for marsh and mangrove vegetation (0.1 to 0.4 meters above mean sea level) [27, 28]. We defined these elevation contours using 30 meter digital elevation models from 1990. To minimize the effect of potential error in those models, we used exactly the same marsh boundaries area again in 2010. In addition to classifying mangroves and salt marsh during the two time periods, eight other coarse land cover classes were classified within the study area in order to improve the classification accuracy. upland (primarily grasses and forbs at elevations above our upper salt marsh boundary), bare/fallow land, forest, beach, urban, tidal flats, other wetlands (primarily non-tidal grasses and forbs), and submerged habitat (“water”).

The TM imagery was transformed using the Tasseled Cap transformation. Initially developed by Kauth and Thomas [29] and later refined for application to TM imagery by Crist and Cicone [30], the Tasseled Cap transformation is set of linear combinations that reduces the spectral data into a new set of bands; the approach is similar in nature to other data reduction techniques such as Principle Components Analysis (PCA). The first three bands of Tasseled Cap reduced data represent the brightness of the image, associated with soil characteristics; the greenness of the image, associated with vegetation; and the wetness of the image, associated with soil moisture [31]. The three brightness, greenness, and wetness bands typically capture over 95% of the variation in the data [31].

Several classification methods were initially explored using the training data. Following numerous classifications and qualitative assessments, we elected to perform classifications through the use of Artificial Neural Networks (ANNs) in order to obtain regional-level classification at a relatively moderate resolution. ANNs have been increasingly used for classifying land cover and frequently outperform traditional classifiers [32]. ANNs also offer several advantages over traditional classification algorithms including flexibility, lack of parametric assumptions, and the ability to handle non-linear and noisy relationships [33, 34]. These advantages allowed us to utilize the information provided by both the spectral TM bands as well as the Tasseled Cap transformed bands. More specifically, the ANNs being utilized were forward-feed, backward propagating, multi-layer perceptrons with a single hidden layer; a common ANN model for the classification of remotely sensed imagery [32]. The ANN classification method was applied to a 9-band image stack of Landsat-5 TM bands 1–5, 7, and Tasseled Cap brightness, greenness, and wetness bands. All image processing was performed in ENVI 4.8 (Excelis Visual Information Systems, Boulder, CO).

Error quantification was performed for both resulting land cover classifications. Initial sample size determination was calculated using a multinomial distribution as described by Congalton and Green [35]. The results from this calculation, assuming a desired precision of 5%, yielded a necessary sample size of n = 633, or 57 samples per class. However, based on the large study area size, we expected that the two cover types of interest (salt marsh and mangrove) would be relatively small proportions of the overall classification area. With this in mind, we increased the sample sizes for each land cover class to 100. The NAPP (1989) and NAIP (2010) DOQQs were also used to assess the classification accuracy; there was no overlap between the training and error assessment samples. Sampling was performed on separate random sample of 3 x 3 clusters to avoid issues related to horizontal precision that could arise on a per-pixel basis [36]. The moderate spatial resolution of this approach was necessary in order to generate estimates of land cover types on the large spatial scale of coastal Texas.

**Fig 2:**
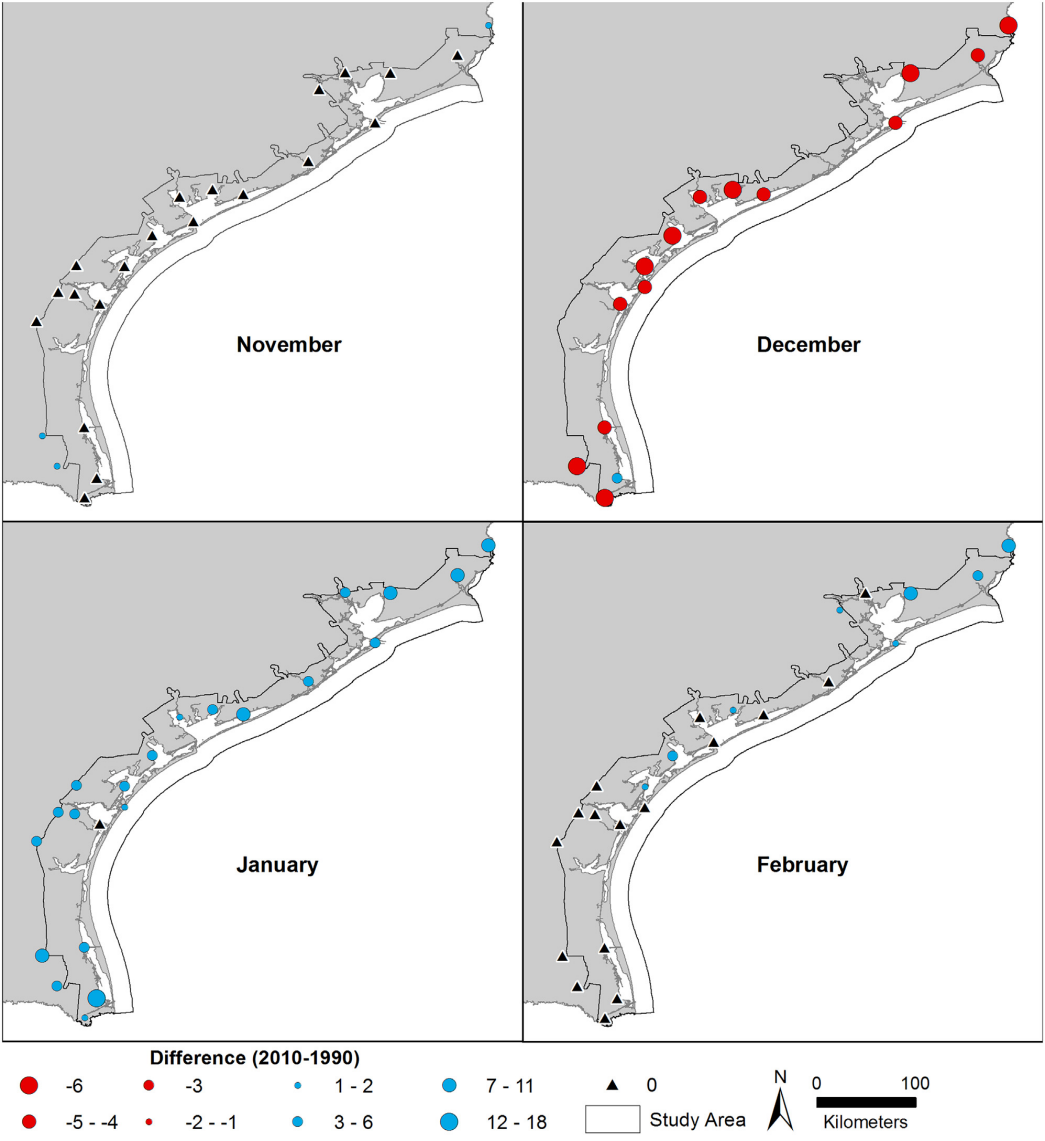
Difference in the number of pre-imagery days with minimum temperatures below 0°C from November—February. Positive values (blue) indicate more freeze days in 2010 compared to 1990 (cooling trend). Negative values (red) indicate more freeze days in 1990 compared to 2010 (warming trend).

To characterize the climatic conditions at each time period, we focused on weather conditions in four months prior to each classification event (November 1989—February 1990 and November 2009—February 2010). Models suggest that mangrove cover on the Gulf Coast is influenced by several winter severity characteristics, including the number of days below freezing (0°C), the number of days below -6.7°C, and the absolute minimum temperature [37]. These winter temperature data were obtained from the NOAA National Climatic Data Center and compared between the two time periods. As shown in Fig 2, weather conditions across the coast were temporally and spatially variable. On balance, the winter severity characteristics in November and February were similar between classification periods. December was colder in 1989, with four or more additional days below freezing at most sites, relative to 2009. In January, there were more days below freezing in 2010 than in 1990 (Fig 2). However, minimum temperatures in 1989–1990 were lower than in 2009–2010, and there were two days in December 1989 with low temperatures less than -6.7°C; winter temperatures in 2009–2010 did not cross that severity threshold, as defined by Osland et al. [37] (Table 2).

**Table 2:** The minimum recorded temperature (°C) at weather stations along the Texas coast, with the number of days with minimum temperatures below -6.7°C in parentheses.

Station	December 1989	January 1990	December 2009	January 2010
Port Arthur	-	-	-	-
Galveston Scholes Field	-	-	34	-
Galveston East End	-10.0 (2)	6.7	1.7	-3.3
Freeport	-	3.3	-1.7	-5.5
Rockport	-7.2 (2)	-0.5	-	-
Port Mansfield	-9.4 (2)	4.4	-1.1	-3.3
Brownsville Airport	-8.9 (2)	3.3	-0.5	-1.7
Port Isabel	-8.3 (2)	5.0	-1.1	-

- indicates that data were not available. Weather data were obtained from the NOAA National Climatic Data Center (http://www.ncdc.noaa.gov/).

We used data from the National Oceanic and Atmospheric Administration (http://tidesandcurrents.noaa.gov/sltrends/sltrends.html) to characterize rates of change in relative sea level at eight stations across the Texas coast; these stations corresponded with the weather stations used in Table 2. Relative sea level rose at all eight stations; rates of increase ranged from 1.9 to 6.8 mm/year, with an average of 4.7 ± 1.6 mm/year (Table 3).

**Table 3:** Average annual rates of change in relative sea level at stations on the Texas coast, listed from south to north.

Path-Row	Station	Rate of change in sea level (mm/yr)
24–39	Sabine Pass	5.66 ± 1.07
25–40	Galveston Pier 21	6.39 ± 0.28
25–40	Galveston Pleasure Pier	6.84 ± 0.81
25–40	Freeport	4.35 ± 1.12
26–40	Rockport	5.16 ± 0.67
26–41	Port Mansfield	1.93 ± 0.97
26–41	Padre Island	3.48 ± 0.75
26–42	Port Isabel	3.64 ± 0.44

Rates are calculated from 1965 (or earlier) through 2006. Data are from http://tidesandcurrents.noaa.gov/sltrends/sltrends.html.

## Results

### Classification Accuracy

Overall classification accuracy was 76% (kappa coefficient = 0.75) in 1990 and 69% (kappa coefficient = 0.66) in 2010. Despite the generally low overall classification accuracies, the individual class accuracies for salt marsh and mangrove cover types were appreciably higher. Classifications performed on the 2010 imagery for salt marsh had an overall accuracy of 89.0% (conditional kappa = 0.73), user accuracy of 89.0%, and producer accuracy of 66.4% (Table 4). Overall mangrove classification accuracy in 2010 was 81.0% (conditional kappa = 0.79) with user and producer accuracies of 81.0% and 98.8%, respectively. The two cover types had similar accuracies for classifications performed on the 1990 imagery. Salt marsh had an overall accuracy of 80.0% (conditional kappa = 0.73), user accuracy of 81.0%, and producer accuracy of 75% (Table 4). Overall mangrove classification accuracy in 2010 was 85.0% (conditional kappa = 0.83) with a user accuracy of 85.0% and producer accuracy of 98.8%.

**Table 4:** Confusion matrices depicting the accuracy of coastal Texas land cover classification in 2010 and 1990.

2010	Ground											
Classified	Salt Marsh	Mangrove	Wetland	Tidal Flat	Bare/ Fallow	Beach	Upland	Forest	Urban	Water	Total	User
Salt Marsh	89	0	3	8	0	0	0	0	0	0	100	89.00%
Mangrove	6	81	2	2	0	0	7	0	0	2	100	81.00%
Wetland	27	0	50	0	0	0	11	0	11	1	100	50.00%
Tidal Flat	1	0	0	80	7	5	0	0	7	0	100	80.00%
Bare/Fallow	3	0	0	3	86	1	4	0	3	0	100	86.00%
Beach	0	0	0	2	47	10	8	0	32	1	100	10.00%
Upland	8	0	3	1	0	0	87	0	1	0	100	87.00%
Forest	0	1	0	0	0	0	57	42	0	0	100	42.00%
Urban	0	0	0	10	13	3	4	0	68	2	100	68.00%
Water	0	0	0	0	0	0	0	0	0	100	100	100.00%
Total	134	82	58	106	153	19	178	42	122	106	1000	
Producer	66.42%	98.78%	86.21%	75.47%	56.21%	52.63%	48.88%	100.00%	55.74%	94.34%		
1990	Ground											
Classified	Salt Marsh	Mangrove	Wetland	Tidal Flat	Bare/ Fallow	Beach	Upland	Forest	Urban	Water	Total	User
Salt Marsh	81	0	4	13	1	1	0	0	0	0	100	81.00%
Mangrove	5	85	0	5	0	1	0	0	1	3	100	85.00%
Wetland	21	1	45	1	8	0	22	0	0	2	100	45.00%
Tidal Flat	0	0	0	81	6	10	2	0	1	0	100	81.00%
Bare/Fallow	0	0	0	0	91	3	6	0	0	0	100	91.00%
Beach	1	0	0	1	12	79	0	0	7	0	100	79.00%
Upland	0	0	1	0	6	0	89	0	3	1	100	89.00%
Forest	0	0	4	0	1	0	49	46	0	0	100	46.00%
Urban	0	0	0	16	8	7	2	0	67	0	100	67.00%
Water	0	0	0	0	0	1	0	0	0	99	100	99.00%
Total	108	86	54	117	133	102	170	46	79	105	1000	
Producer	75.00%	98.84%	83.33%	69.23%	68.42%	77.45%	52.35%	100.00%	84.81%	94.29%		

### Land Cover Changes

**Fig 3:**
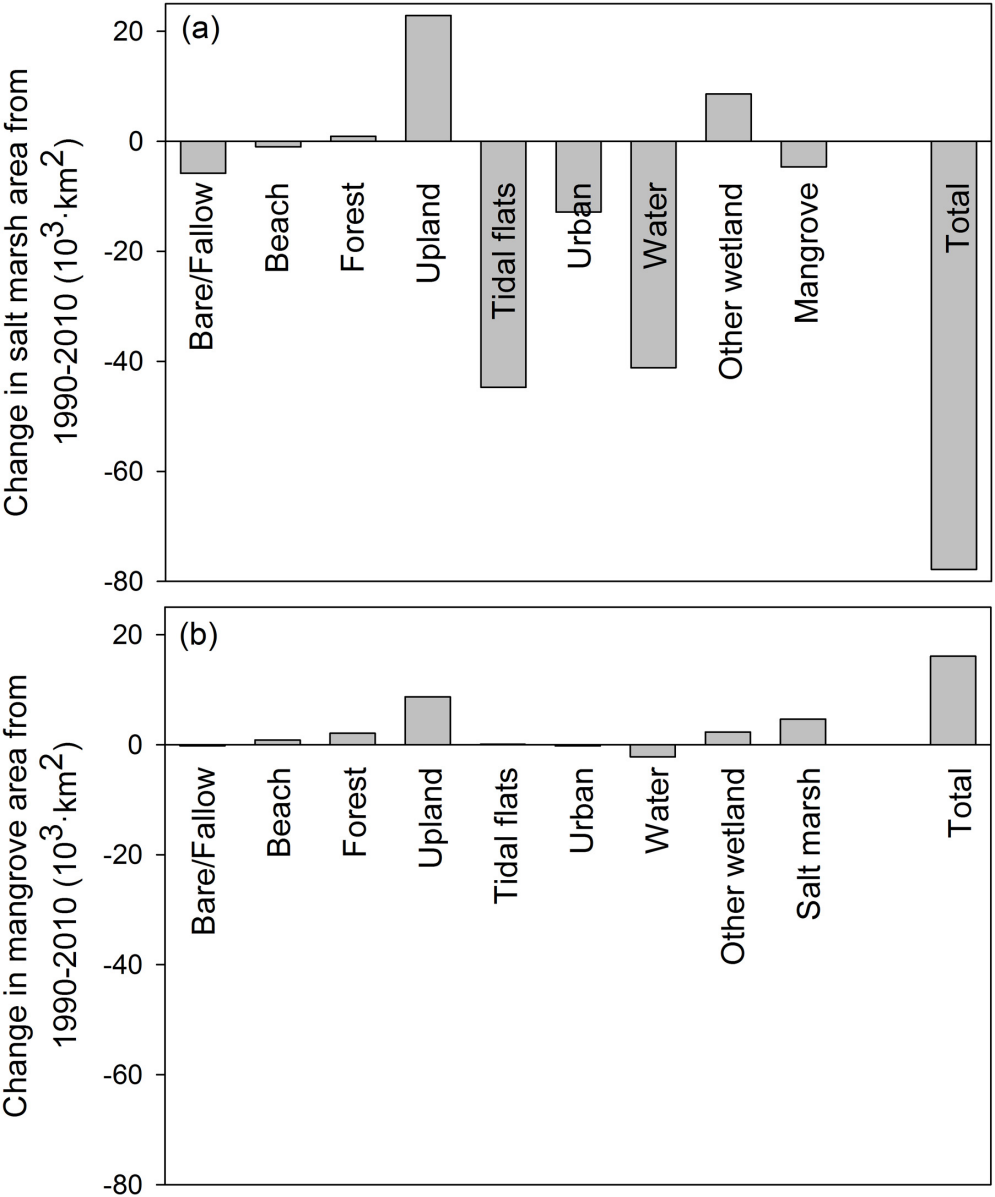
Net change in (a) salt marsh and (b) mangrove area. Changes are broken down by land cover type and are summed across the entire Texas coast from 1990 to 2010.

When focusing on our specific habitats of interest—salt marshes and mangroves—we detected substantial changes in total area. Salt marshes decreased from 318.27 to 240.44 km^2^, a net loss of 77.82 km^2^, or -24% of the 1990 salt marsh area (S1 Table). Salt marshes lost the most area through conversion to tidal flats (-44.75 km^2^) or water (-41.16 km^2^), but only -4.66 km^2^ due to mangrove expansion (Fig 3a). These losses were only partially offset by expansion of +22.87 km^2^ of salt marshes into upland habitat. Mangroves increased from 21.81 to 37.90 km^2^, a net gain of 16.09 km^2^, or +74% of the 1990 mangrove area. Most of the mangrove gain was a result of encroachment on upland (+8.71 km^2^) or salt marsh (+4.66 km^2^); only a small portion (-2.21 km^2^) was lost through conversion to water (Fig 3b).

**Fig 4:**
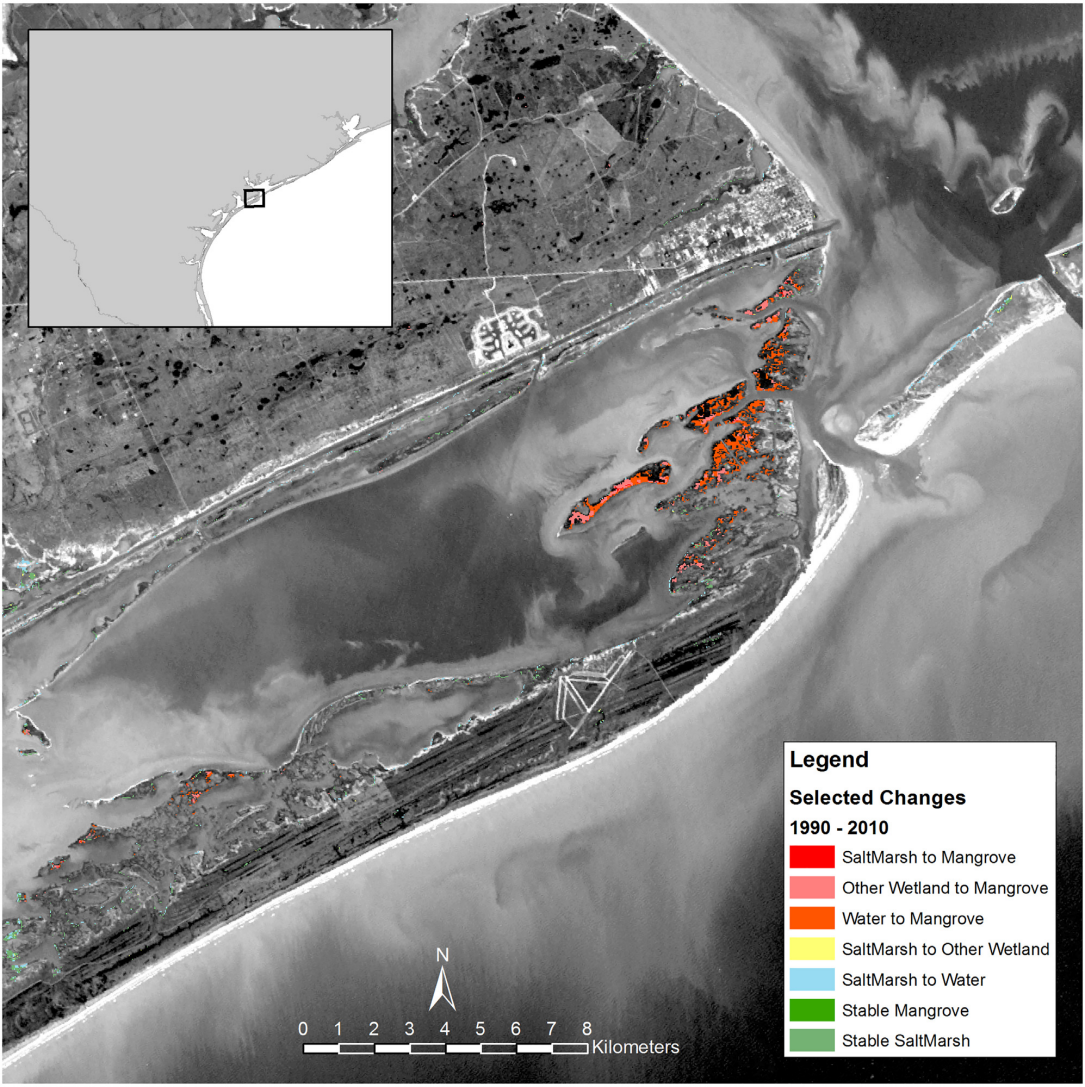
Change in land cover type from 1990 to 2010 near Port O’Connor, Texas.

**Fig 5:**
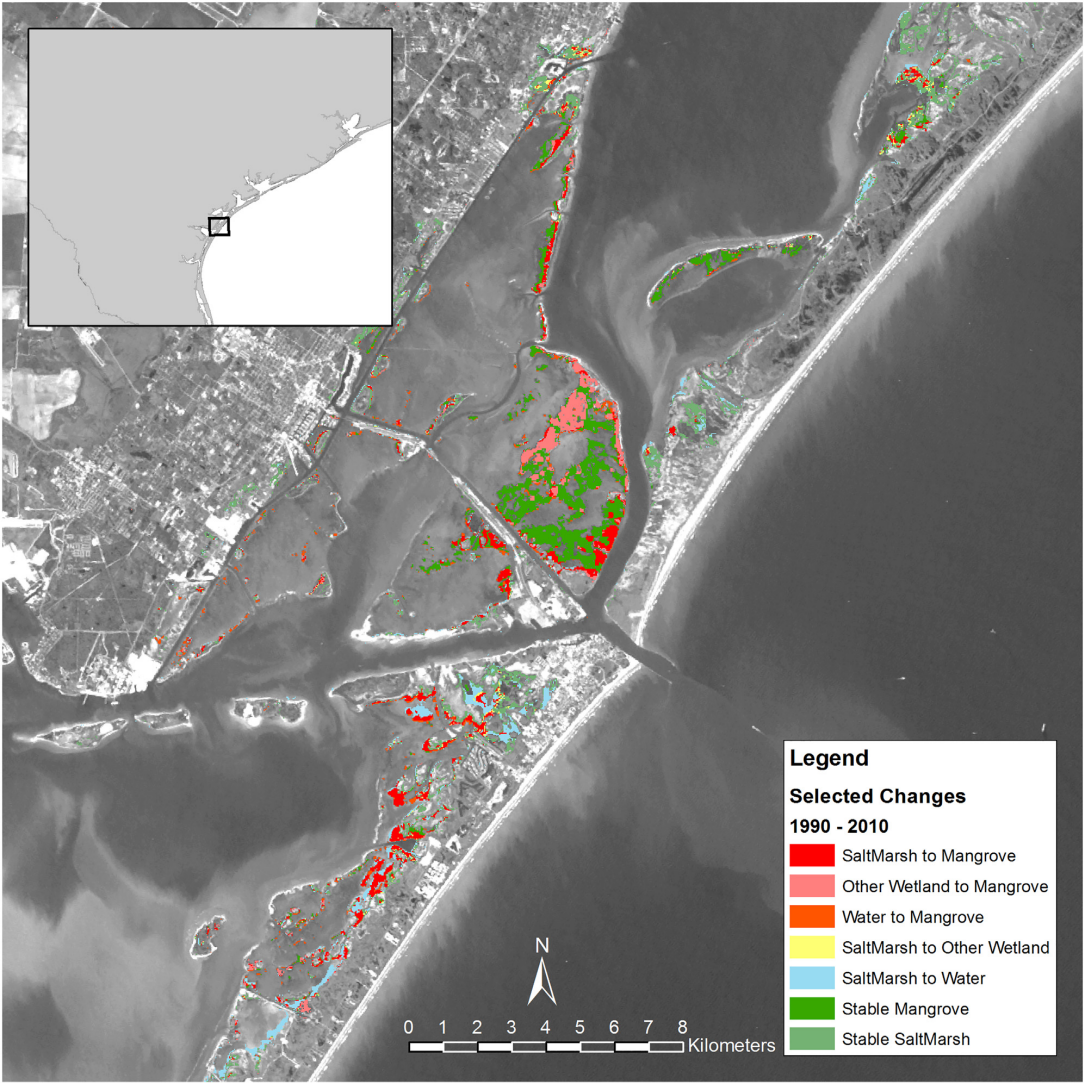
Change in land cover type from 1990 to 2010 near Port Aransas, Texas.

**Fig 6:**
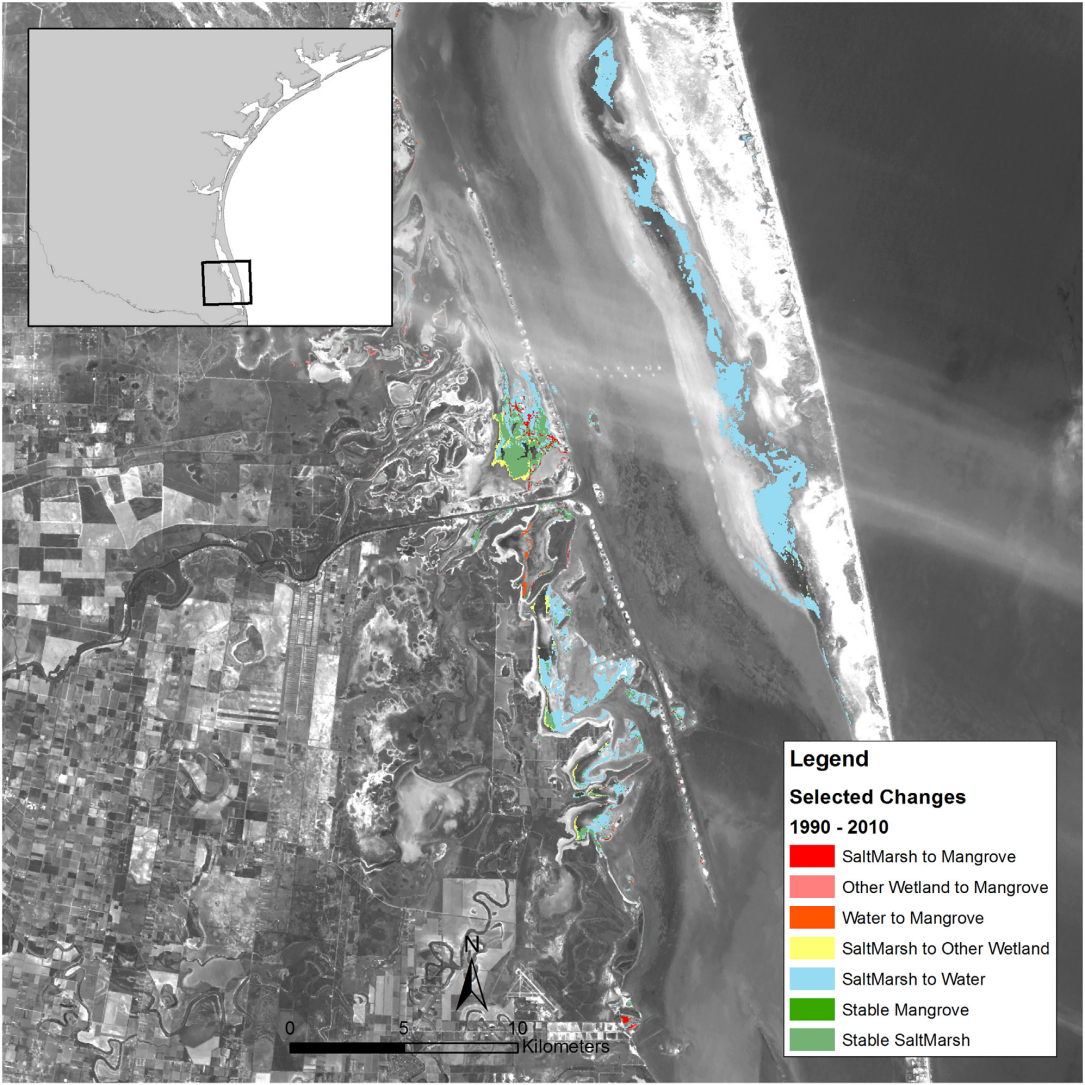
Change in land cover type from 1990 to 2010 near South Padre, Texas.

Based on previous studies [16, 19, 20], we identified three of the areas on the Texas coast where mangroves have been expanding, and then mapped the land cover changes in each of those areas. In the Espiritu Santu Bay near Port O’Connor, most of the new mangrove area was converted from submerged habitat, some of which may have been salt marsh at high tide in 1990 (Fig 4). In contrast, at the state-level, there was a small though detectable net loss of mangrove habitat to submerged habitats (Fig 3b). In the Harbor Island/Port Aransas/Mustang Island area, there was a large stable mangrove population (Fig 5). Most of the gain in mangroves in this area was from salt marsh and other wetlands. A substantial amount of salt marsh loss via conversion to water habitat also occurred on Mustang Island. In the South Padre area, the absolute increase in mangrove area was comparatively small; most mangrove gain in this area was converted from salt marsh (Fig 6). There were also several small areas of salt marsh that were converted to water habitat in the South Padre area.

## Discussion

Our results indicate that mangroves in Texas are expanding and, in some instances, displacing salt marshes. This conclusion supports the numerous localized accounts of mangrove expansion throughout the Gulf coast [14, 16–20, 38] and east coast of Florida [39]. However, our analysis also reveals that over the twenty-year period from 1990 to 2010, there was no large-scale replacement of salt marsh by mangroves along the Texas coast. Our study added another important insight. mangrove expansion is not the only driver of salt marsh loss, and may not even be the primary driver of salt marsh loss in some parts of the marsh-mangrove ecotone.

Based on the classification methods implemented for our analysis, mangrove area is an order of magnitude smaller (21–38 km^2^) than that of salt marshes (240–318 km^2^). We concede that mangrove extent may have been underestimated because the signature of subcanopy water may have swamped mangrove signals, especially in low density stands. However, coastal salt marshes have been remotely sensed and quantified by numerous studies with high levels of accuracy, typically 70% and better [40–44]. The Landsat Thematic Mapper (TM) sensor is reliable for this type of wetland detection and classification [41]. Mangroves have also been delineated with high accuracy using medium resolution imagery. For example, Butera [40] achieved 87% accuracy in black mangrove classification using Landsat MSS in Florida. Gao [45] achieved 95% accuracy using Landsat TM imagery, yielding more accurate results than estimates using higher resolution 20 m SPOT imagery. Other research focused on mangrove classification has consistently realized 70%+ classification accuracy using Landsat TM imagery [46–48]. The history of successful and accurate classification of salt marsh and mangrove environments demonstrates the ability to remotely sense these specific cover types at a regional scale, using medium resolution (30 m), multi-spectral imagery. Therefore, despite our potential underestimation of mangrove cover, our classification errors were relatively low, and our analysis strongly suggests that the state-level ratio of mangrove to salt marsh area was very small. Therefore, recent mangrove expansion has remained a relatively local, rather than a regional issue in the western Gulf of Mexico. Salt marsh loss, on the other hand, was widespread throughout the study region.

Localized mangrove expansion in Texas coastal ecosystems is mirrored in other marsh-mangrove ecotones around the world [1, 39, 49, 50]. The ecological, hydrological, and geomorphological implications of these expansions include changes to fishery support, carbon sequestration rates, and shoreline stabilization [19, 51–53], though many effects still need to be better quantified. Many further questions remain regarding the implications of mangrove expansion for regional economies [54].

The role of freezing events—specifically, a decrease in their frequency and intensity—has been widely implicated in the local and regional expansion of mangrove stands [37, 39, 55]. Mangroves are sensitive to cold temperatures, and *Avicennia germinans* experiences partial or full mortality in laboratory experiments if air or soil temperature is less than -6.5°C for 24 hours [56]. In the field, temperatures less than -4°C can cause mangrove mortality [39], and models predict that temperatures less than -6.7°C are necessary to cause substantial mangrove mortality [37]. Correlative and modeling studies have clearly demonstrated that mangrove cover is lower in years with more days below freezing [37, 39]; lowered mangrove cover may persist for several years following a hard freeze [39, 55]. In fact, a severe freeze event in 1983 temporarily reduced mangrove density in some Gulf populations by over 90% [57, 58]. There is, as of yet, no strong evidence of a dramatic decrease in freezing event frequency or severity in Texas over the last 100 years [59]. However, most climate change models suggest a future reduction in the frequency, severity, and length of freezing events in the Gulf of Mexico [21]. When local mangrove responses are integrated with these climate projections, models predict regional-scale replacement of salt marshes by mangroves within 100 years [37].

Freeze events occurred in the study area in December 1989 and January 2010; in both cases the freezes occurred 3–5 months before our aerial imagery was collected. These events reduced mangrove cover in portions of the study area (J.F. Schalles, pers. comm., [60]). Although some locations recovered quickly within a few months [58, 60], neither survey period had a full growing season to recover. The 1989 freeze was particularly severe, with lower minimum temperatures and more days below the -6.7°C mortality threshold (Table 2), and regrowth was likely slower than in 2010. Therefore, we may have underestimated mangrove cover in 1990 and subsequently overestimated the amount of mangrove expansion over the study period. However, relative to marsh area, the mangrove area was very small, suggesting that other drivers of salt marsh loss shaped the coastal landscape at a large spatial scale.

Based on our analysis, sea level rise was clearly though indirectly implicated as a driver of salt marsh loss, as indicated by the conversion of salt marsh to water habitat. Sea level is widely acknowledged as rising across the Gulf Coast of the United States at a relatively rapid rate, driven by local and eustatic forces [61]. Sea level rise is a known driver of salt marsh loss, particularly when coastal development limits the potential for upland migration, causing a phenomenon known as coastal squeeze. Even moderate sea level rise can therefore cause extensive coastal wetland loss [62]. Accordingly, in our study, the majority of the area lost from salt marsh was converted to water (subtidal habitat) or tidal mudflats. This estimate of salt marsh loss due to relative sea level rise is probably conservative, since tides during the survey period were slightly higher in 1990 than in 2010. Some areas of salt marsh may have been inundated in 1990, leading us to under-calculate the areal extent of marsh and thus underestimate the loss of marsh to subtidal habitat from 1990–2010.

The implications of relative sea level rise for coastal wetland distribution and vegetation type are complex, given the potential differences between mangroves and salt marshes in terms of resilience to sea level rise [38]. In some regions, mangrove stands have higher accretion rates than marshes [63, 64], which can subsequently lead to accelerated expansion of mangroves [65]. These differences in marsh and mangrove accretion rates are well documented in Australia [63, 64]; accretion rates in marsh and mangrove stands in the Gulf of Mexico are more variable and not as clearly linked to vegetation type [27]. Our analysis detected relatively little mangrove conversion to subtidal (water or tidal flat) habitat at a state-level spatial scale, suggesting that marshes have been more severely affected by near-term sea level rise on the Texas coast. However, both marshes and mangroves are potentially vulnerable to inundation; neither habitat type has accretion rates that are consistently above the recent rate of relative sea level rise on the Gulf Coast, which sometimes exceeds 6 mm/year [27, 61].

The dynamic changes in the relative distribution of salt marshes and mangroves is further complicated by the potential influence of changes in freshwater supply. Freshwater is a carefully and contentiously managed resource in most estuaries [66–68], and increases in freshwater supply may increase mangrove encroachment rates [38]. Some species of marsh vegetation are more tolerant than mangroves to the hypersaline conditions that are typical of the southern Texas Gulf Coast [23]. In these types of arid environments, a small change in rainfall may result in a dramatic shift in foundation plant species cover [69]. Rainfall and resultant estuarine salinity on the Texas coast have high interannual variability [20]. However, there was an El Niño event, associated with above-average rainfall, within two years prior to each of our sampling periods [70, 71], suggesting that coarse variation in freshwater supply was not the primary driver of the state-level changes in the coastal landscape from 1990 to 2010.

Our study revealed a recent state change in the coastal landscape of Texas. Substantial areas of salt marsh have been submerged over the last 20 years, with only partial replacement by mangrove expansion. Recent models suggest that mangrove expansion will continue, leading to nearly complete mangrove replacement of salt marshes on the Texas coast within the next 100 years [37]. Alternatively, given the complex interplay between accretion rates, cold tolerance, and carbon dioxide response, the Texas coastal landscape may oscillate between alternate stable grass- and mangrove-states [9]. Regardless of the climate-related trajectory of mangrove expansion, our analysis showed that salt marsh loss is extensive, and is not exclusively linked to mangrove expansion. Relative sea level rise is also a likely cause of irreversible marsh loss in the western Gulf of Mexico. The rate of coastal wetland loss highlights the importance of avoiding coastal squeeze by integrating upland migration “escape” routes into land management and restoration practices [64].

## Supporting Information

S1 Table Change matrix indicating the area of coastal zone land cover changes in Texas from 1990–2010 in km^2^. (DOCX)
