# Supplementary material for: The Contribution of Mangrove Expansion to Salt Marsh Loss on the Texas Gulf Coast
Source: PLoS One. 2015 May 6;10(5):e0125404. doi: 10.1371/journal.pone.0125404 (PMC4422646; doi:10.1371/journal.pone.0125404)
Supplement: S1 Table — (DOCX) [file pone.0125404.s001.docx]

**Table S1: Change matrix indicating the area of coastal zone land cover changes in Texas from 1990 – 2010 in 1000*km^2^.**

|  | 2010 Land Cover | | | | | | | | | | |
| --- | --- | --- | --- | --- | --- | --- | --- | --- | --- | --- | --- |
| 1990 Land Cover | Bare/Fallow | Beach | Forest | Mangrove | Upland | Salt Marsh | Tidal Flats | Urban | Water | Other wetland | Total |
| Bare/Fallow | 2064.27 | 134.05 | 76.17 | 0.07 | 462.31 | 16.85 | 55.1 | 116.59 | 29.93 | 303.56 | 3258.9 |
| Beach | 144.85 | 161.96 | 71.38 | 0.92 | 105.55 | 3.48 | 211.96 | 66.5 | 44.55 | 52.52 | 863.66 |
| Forest | 210.21 | 4.63 | 252.65 | 2.12 | 716.57 | 1.87 | 2.14 | 71.87 | 15.33 | 490.73 | 1768.11 |
| Mangrove | 0.26 | 0.06 | 0.03 | 6.96 | 0.2 | 1.37 | 1.69 | 0.63 | 6.89 | 3.71 | 21.81 |
| Upland | 1240.27 | 237.78 | 1147.61 | 8.91 | 2472.83 | 29.83 | 71.25 | 283.42 | 105.44 | 1326.43 | 6923.77 |
| Salt Marsh | 22.64 | 4.48 | 0.95 | 6.03 | 6.96 | 132.08 | 49.88 | 17.38 | 64.59 | 13.27 | 318.27 |
| Tidal Flats | 27.66 | 7.78 | 0.62 | 1.77 | 6.58 | 5.13 | 437.22 | 22.76 | 61.42 | 16.83 | 587.78 |
| Urban | 110.64 | 39.71 | 9.92 | 0.41 | 66.74 | 4.53 | 113.71 | 313.2 | 36.02 | 125.89 | 820.76 |
| Water | 49.83 | 26.39 | 0.96 | 4.68 | 16.47 | 23.43 | 103.92 | 39.19 | 6701.68 | 103.4 | 7069.96 |
| Other wetland | 823 | 32.38 | 59.94 | 6.03 | 608.5 | 21.87 | 59.26 | 204.34 | 206.3 | 1308.61 | 3330.22 |
| Total | 4693.61 | 649.22 | 1620.22 | 37.9 | 4462.71 | 240.44 | 1106.13 | 1135.87 | 7272.16 | 3744.96 | 24963.23 |
